# Permeation of β-Lactamase Inhibitors through the General Porins of Gram-Negative Bacteria

**DOI:** 10.3390/molecules25235747

**Published:** 2020-12-05

**Authors:** Alessandro Pira, Mariano Andrea Scorciapino, Igor V. Bodrenko, Andrea Bosin, Silvia Acosta-Gutiérrez, Matteo Ceccarelli

**Affiliations:** 1 Department of Physics, University of Cagliari, Cittadella Universitaria di Monserrato, 09042 Monserrato, Italy; alessandro.pira@dsf.unica.it (A.P.); andrea.bosin@dsf.unica.it (A.B.); 2 Department of Chemical and Geological Sciences, University of Cagliari, Cittadella Universitaria di Monserrato, 09042 Monserrato, Italy; scorciapino@unica.it; 3 CNR/IOM Sezione di Cagliari, Cittadella Universitaria, 09042 Monserrato, Italy; igor.bodrenko@cnr.it; 4 Department of Chemistry, University College, London WC1E 6BT, UK; s.gutierrez@ucl.ac.uk

**Keywords:** beta-lactamase inhibitors, molecular dynamics simulations, Gram-negative bacteria, bacterial porins, free energy surface, permeation of small molecules

## Abstract

Modern medicine relies upon antibiotics, but we have arrived to the point where our inability to come up with new effective molecules against resistant pathogens, together with the declining private investment, is resulting in the number of untreatable infections increasing worldwide at worrying pace. Among other pathogens, widely recognized institutions have indicated Gram-negative bacteria as particularly challenging, due to the presence of the outer membrane. The very first step in the action of every antibiotic or adjuvant is the permeation through this membrane, with small hydrophilic drugs usually crossing through protein channels. Thus, a detailed understanding of their properties at a molecular level is crucial. By making use of Molecular Dynamics simulations, we compared the two main porins of four members of the *Enterobacteriaceae* family, and, in this paper, we show their shared geometrical and electrostatic characteristics. Then, we used metadynamics simulations to reconstruct the free energy for permeation of selected diazobicyclooctans through OmpF. We demonstrate how porins features are coupled to those of the translocating species, modulating their passive permeation. In particular, we show that the minimal projection area of a molecule is a better descriptor than its molecular mass or the volume. Together with the magnitude and orientation of the electric dipole moment, these are the crucial parameters to gain an efficient compensation between the entropic and enthalpic contributions to the free energy barrier required for permeation. Our results confirm the possibility to predict the permeability of molecules through porins by using a few molecular parameters and bolster the general model according to which the free energy increase is mostly due to the decrease of conformational entropy, and this can be compensated by a favorable alignment of the electric dipole with respect to the channel intrinsic electric field.

## 1. Introduction

Antibiotics have been regarded as magic bullets to treat infections for almost the entire 20th century. Modern medicine relies upon them, but due to their spread misuse, we have arrived to the point where almost all antibacterial drugs developed so far have been followed by detection of resistance [1,2]. Resistance is related to bacteria innate evolution, and, briefly, it is a condition by which susceptibility to the antibiotic is reduced. This is accelerated by the extensive use of antibiotics in human and human-related activities, meaning that we will always need new antibiotics. However, we are facing a real global challenge for modern medicine, as our inability to come up with new effective molecules [3], together with the declining private investment [1], results in the number of untreatable infections increasing worldwide at worrying pace.

Many fundamental gaps need to be filled in our knowledge about antibiotics. The mode of action, the penetration pathway, and the mechanisms adopted by the pathogen to degrade or pump drugs out are just a few general examples. High-throughput screening protocols are looked with great hope as a way to identify novel drug scaffolds, but these protocols need to be fed with proper selection rules, otherwise the risk is high that they can be biased [4,5]. Along this direction, some preliminary “rules for good antibiotics” were recently proposed [6,7]. Artificial Intelligence has been recently proposed as a new approach to provide new molecules [8], although this does not provide any new knowledge about the mode of action. The problem is urgent, since the pipeline is virtually empty of new scaffolds. For this reason, alongside with the research of new antibiotics, also adjuvants are highly requested to improve the efficacy of the already available antibacterials [9,10].

Among other pathogenic microorganisms, which certainly deserve much attention, widely recognized institutions have indicated Gram-negative bacteria as particularly challenging [2,11,12,13]. All bacteria have an inner plasma-membrane, with the Gram-negative ones characterized by the presence of an additional outer membrane, which provides the cell with an intrinsic and very effective resistance mechanism. The outer membrane works with a variety of membrane proteins and protein complexes to maintain a strict control on the entry of needed species, as well as toxic compounds, with its overall permeability playing an essential role in the susceptibility to the antibiotics [14,15]. While the permeation of small hydrophobic molecules through the membrane inner core is known [16], Gram-negative bacteria are usually not susceptible to hydrophobic antibiotics like actinomycin D, macrolides or novobiocin [17]. Further, the majority of small hydrophilic antibacterials has been recognized to make use of pore-forming proteins to access the periplasmic space [14,15,16,17,18]. It is not surprising to find porins mutations as marker of numerous resistant strains [15,16,17,18,19]. Moreover, the overexpression of multidrug efflux pumps has been increasingly found to be associated to antibiotic resistance [20], and of course, resistance can be also obtained by modification of the drug target and by the expression of degradation enzymes [21]. However, the very first step in the action of every antibiotic is the permeation through the outer membrane; thus, a detailed understanding of porins’ properties at a molecular level is crucial to elucidate their filtering role and to make profitable the use of this information for antibiotic optimization [22]. The abovementioned problems are faced also by numerous classes of adjuvants like all the hydrophilic inhibitors of the degradative enzymes or efflux pumps, making the translocation problem absolutely general.

Porins are typically arranged either as homotrimers or monomers, with a basic β-barrel folding that forms a transmembrane water-filled pore. While species like *Pseudomonas aeruginosa* and *Acinetobacter baummanii* are characterized by a variety of rather small porins that are specific for the uptake of the different nutrients [23,24,25], the *Enterobacteriaceae* express two principal trimeric porins. These are classified as nonspecific general porins, since they allow the passage of ions and metabolites up to 600 Da with no clear specificity [26]. Since *Escherichia coli* is usually taken as prototypical example for enteric Gram-negative bacteria, the literature is more extended than about other species. In addition, in order to find possible general rules for protein channels permeation, we preferred to focus on general porins, leaving the specific ones to a next stage. In the case of *E. coli*, the major porins are OmpF and OmpC. They share remarkable similarities in their amino acid sequence and three-dimensional structure, with OmpC being narrower than OmpF. Moreover, the level of expression of these two porins differs with varying growth conditions, leading to the conclusion that pore size is key to channel permeability [27]. However, both experimental and computational evidence show the strong role played by internal electrostatics on permeation properties [28,29]. Experiments showed remarkably different diffusion characteristics towards different solutes [17,28,30,31]. Despite considerable efforts, no evident strong correlation was found between the rates of permeation and any of the invoked molecular properties, such as the size (volume or mass), net charge, hydrophobicity, and number of H-bond donors/acceptors, to name a few [28,29,30,32,33,34,35,36,37,38]. For example, it has been recently suggested that the size limit of 600 Da should be better defined by using other parameters [39].

OmpF and OmpC are homotrimers formed by 16-strand β-barrels. The size of the lumen is determined by the loop L3, which folds into the channel and forms a restricted portion almost halfway through the pore. This is usually referred to as the constriction region (CR), which separates the extracellular vestibule from the periplasmic one (EV and PV, respectively) in an overall hourglass shape. The loop L3 is characterized by several negatively charged residues facing a series of aligned basic residues of the β-barrel, the so-called basic ladder, which results in a remarkable transversal electrostatic potential gradient [40,41]. The general electrostatics of the lumen, more than its size, was suggested to be of primary importance [28,33]. For many years, strong interactions between the diffusing species and the amino acid residues of the CR were thought to play a central role, by increasing the probability for the “substrate” to cross the channel and eventually emerge on the periplasmic side [42]. More recently, the idea of strong direct interactions has been questioned [30]. Although the presence of specific binding sites might have some effects on the diffusion rate, these are expected to be only subtle at physiological concentration, with rates being mostly determined by the general physicochemical properties of the molecule.

Recently, we performed an atom-level computational comparative study [43] on several OmpC mutants by taking the rare opportunity of serial mutations observed in successive *E. coli* resistant clinical strains isolated from a patient treated over two years with cephalosporins, carbapenems, and fluoroquinolones [33,44]. Interestingly, the occurred mutations did not result in significant changes in the pore size or ion conductivity. Both the loop L3 and the basic ladder were found to be highly conserved. We exploited the natural presence of the water molecules inside these general porins to probe the channel’s internal electric field (EF), by calculating the electric polarization of the solvent at different depth inside the channel. The main differences were not in the CR but surprisingly found in the EV at the interface with the CR, which was referred to as the pre-orientation region (POR) [43]. More recently, we proposed a more accurate computational method to quantitatively determine the macroscopic EF inside these nano-sized water-filled channels, which can also be extended to the external surface of proteins or other biological systems and to water-filled cavities [45]. A series of works focused on β-lactam antibiotics [43,46,47,48] indicated that, during substrate translocation, there is a compensation between entropic reduction, due to the size-confinement of the CR, and the enthalpy gain coming from the favorable alignment of its electric dipole with respect to the channel internal EF. In addition, the field at the POR is extremely important because it can drive the dipolar drug to assume a given orientation before entering the CR, thereby facilitating the overall translocation process or making it more difficult.

In this work, by making use of Molecular Dynamics (MD) simulations, we first performed the structural comparison of the two main porins of four different members of the *Enterobacteriaceae* family, namely, *E. coli*, *Enterobacter aerogenes*, *Enterobacter cloacae*, and *Klebsiella pneumoniae*, in order to verify whether the electrostatic fingerprint of OmpF and OmpC can be actually taken as a model for other species of the same family. Then, we used biased Metadynamics simulations (metaMD) to reconstruct the translocation free energy of selected β-lactamase inhibitors through OmpF. All the selected inhibitors are avibactam derivatives, belonging to the class of diazobicyclooctans. Thus, they are not β-lactams, and as it has been recently shown by using other scaffolds, [49,50] they will contribute to understand to a more general level the process of small-molecule permeation through Gram-negative general porins.

## 2. Results and Discussion

### 2.1. The Electrostatic Fingerprint of the OmpF and OmpC Orthologues

The first three-dimensional structure of OmpF from *E. coli* at atomic level was published in 1992 [51], OmpC in 2006 [52]. OmpF and OmpC are commonly assumed as prototypical examples for outer membrane bacterial general porins both in computational and experimental studies. To demonstrate it, we took the structure of OmpF/OmpC orthologs from other species of the *Enterobacteriaceae*: Omp35/Omp36, OmpE35/OmpE36, and OmpK35/OmpK36, respectively, from *E. aerogenes*, *E. cloacae*, and *K. pneumoniae* [7]. We compared the main structural features, namely the amino acid sequence, the internal pore radius, the distribution of charged residues inside the lumen, and the internal electric field, as obtained from MD simulations.

The structures obtained by X-ray diffraction show the same trimeric quaternary structure and share the same β-barrel architecture of each monomer, comprising 16 strands linked by short turns on the intracellular side and longer loops on the extracellular one (Supplementary Materials Figures S2 and S3). The loop L3 is folded inside the lumen in each monomer, in all the crystallographic structures, and is responsible for the characteristic internal hourglass shape.

The alignment of all structures is shown in Supplementary Materials Figures S4 and S5. The most evident differences are found in the external loops, in particular, the insertion in loop L1 of OmpF and in loop L5 of Omp36. Conversely, the loop L3 is found to be conserved, notably the negatively charged residues D113, E117, and D121 in the case of OmpF, and the residues D105, E109, and D113 in the case of OmpC. These are very important because, located in the CR, they face a series of aligned positively charged residues on the other side of the channel, the so-called basic ladder. These are also conserved in the orthologs investigated here, corresponding to the residues K16, R42, R82, and R132 in the case of OmpF, and the residues K16, R37, R74, and R124 in the case of OmpC. Such striking charge segregation is responsible for the strong EF at the CR [45].

In a previous work [43], the structural alignment between OmpF and OmpC showed very important differences outside the CR. In particular, in the EV, the K80 and R167 of OmpF correspond to W72 and L173 in OmpC. These mutations are responsible for the striking different EF in the so-called pre-orientation region [43,45]. These positions are located right at the entrance of the CR, and the related difference in the EF was shown to have a large impact on the translocation free-energy of a number of antibiotics [43,46,47,48]. The structure alignments in Supplementary Materials Figures S4 and S5 show that these specific differences are conserved in the OmpF and OmpC orthologs.

**Figure 1:**
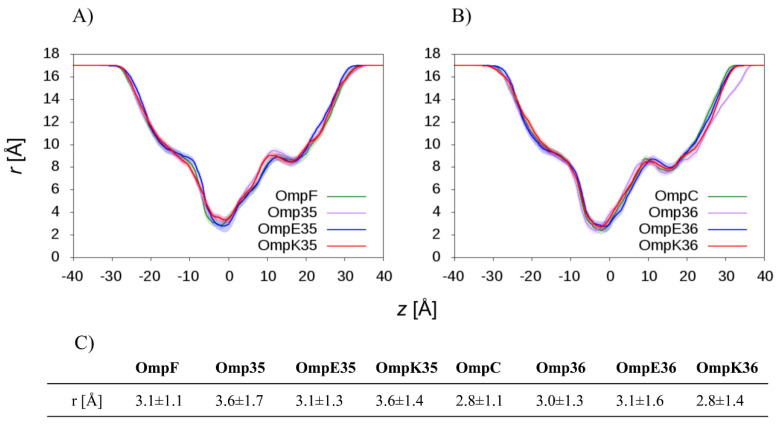
Channel radius profile (time-average) as a function of the *z*-coordinate (aligned with the main axis of the channel) in the case of (**A**) OmpF and its orthologs, and (**B**) OmpC and its orthologs. The extracellular side correspond to positive values of z, the periplasmic side to the negative ones. The shaded area represents the standard deviation over the entire Molecular Dynamics (MD) trajectory. (**C**) Values of the minimum radius (observed at the constriction region (CR)) and the corresponding standard deviation are reported.

The main energy barrier for substrate translocation is expected to be of steric nature; thus, a cross-section profile of the protein channel is of primary importance. We calculated the average radius of the lumen as a function of the *z* coordinate, here chosen to be aligned with the main axis of the channel. The calculation was performed on the last 300 ns of our MD trajectories (400 ns), and results are reported as time averages. Supplementary Materials Figure S6 shows that all porins have an RMSD stable over the entire trajectory and not higher than 2.0 Å from the starting X-ray structure. MD simulations were run also with different potassium chloride concentrations (from 0.2 to 1.0 M) to investigate the role of the ionic strength. Radius profile of the porins did not show any significant difference, although OmpC orthologs, on average, showed slightly shorter values. The ionic strength appeared not to influence the intrinsic structure of these porins within the investigated range. Figure 1 shows the results obtained at 200 mm KCl.

All the investigated porins are characterized by a net negative charge and have been reported to be slightly cation selective [7]. The selectivity to ions is defined as the ratio between the permeability to positive over negative ions, PK+PCl−. Results obtained from experiments of single-channel electrophysiology [7] are reported in Table 1.

**Figure 2:**
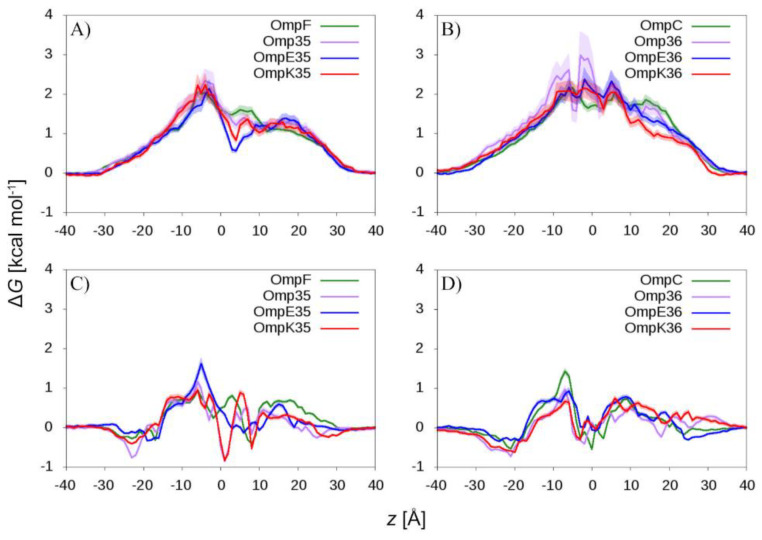
Free energy profile as a function of the *z*-coordinate (i.e., the main axis of the channel) for (**A**,**B**) chloride and (**C**,**D**) potassium ion permeation. Results are shown for (**A**,**C**) OmpF and its orthologs, and (**B**,**D**) OmpC and its orthologs. The extracellular side correspond to positive values of *z*, the periplasmic side to the negative ones. The shaded area represents the standard deviation over the entire MD trajectory.

From a general point of view, the slightly larger negative net charge of OmpC and its orthologs correlates with the higher selectivity for cations than OmpF and its orthologs. However, in order to obtain additional details about structural differences, we calculated the free energy profile of ion permeation from MD simulations. The density of ions was derived as the time average over the three monomers as a function of the *z*-coordinate and compared with that of the bulk solution outside the channel through the following equation:

$$\Delta G\left(z\right)=-k_{B}T\ln\left(\frac{\rho \left(z\right)}{\rho_{bulk}}\right)$$

where ρ is the ion density, $k_{B}$ is the Boltzmann constant, and T the absolute temperature. Results are shown in Figure 2.

In all of these general porins, while free energy oscillates around zero for K^+^, the profile for Cl^−^ is characterized by a wide barrier over almost the entire channel axis, which accounts for the cation selectivity. In OmpF and its orthologs, a minimum is present in the POR, right above the CR, such that, overall, the energy cost for translocation is larger in the case of OmpC and its orthologs. The different profile is attributable to the abovementioned K80W and R167L mutations (OmpF sequence number is used; see the structural alignment). These observations account for the slightly larger cation selectivity of OmpC and its orthologs and bolster the importance of the POR in differentiating the two groups of general porins investigated.

We complemented this analysis and the reported structural alignment with the inspection of the distribution of charged residues along the channel. The channel was divided into five cross-sections along the main axis, namely +16 Å > *z* > +10 Å; +11 Å > *z* > +5 Å; +6 Å > *z* > 0 Å; +1 Å > *z* > −5 Å; and −4 Å > *z* > −10 Å. For each section, the *xy*-distribution of positively and negatively charged residues was obtained from the coordinates recorded along the MD trajectory and their difference calculated. Supplementary Materials Figure S7 shows the results for the eight general porins. The only relevant difference between OmpF and OmpC orthologs in the charged residues distribution is found at level of the POR. A clear spot of positive charge density is found in the former, while the same area of the channel is virtually neutral in the latter. This generates a significant segregation of charges on opposite sides of the lumen in the case of OmpF and its orthologs, which is absent in OmpC and its orthologs. This region, as already mentioned, was already identified [43]. It was shown to be responsible of the pre-orientation of the dipolar substrates when approaching the CR coming from the EV, and of the high affinity of ampicillin [36]. It was shown to be a striking difference between OmpF and OmpC and here it is revealed as a structure characteristic of the entire series orthologs selected among other *Enterobacteriaceae*. The presented results complement the already reported EF calculations for the same general channels [7] and confirm that OmpF and OmpC can be actually used as good models for this kind of general outer membrane channels.

### 2.2. The Permeation Free-Energy of Diazobicyclooctans β-Lactamase Inhibitors through OmpF

**Figure 3:**
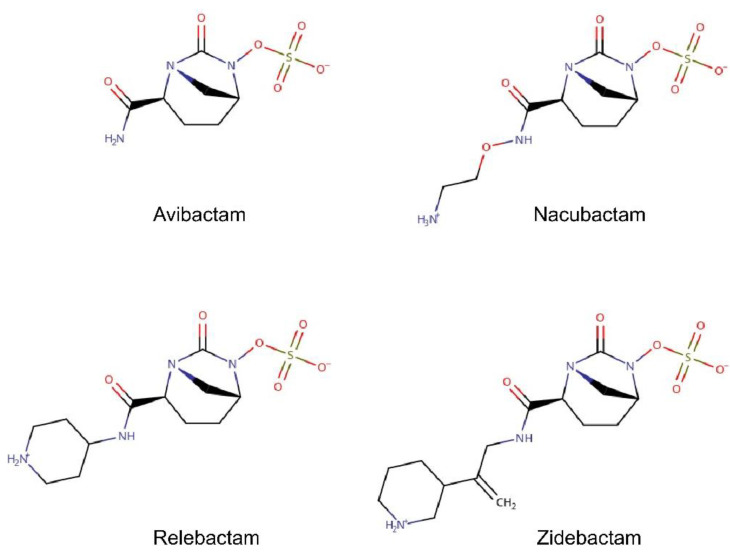
Lewis structure of avibactam and the four selected derivatives investigated in this work.

In a recent work [53], we have already reported the first free-energy surface (FES) for the permeation of avibactam (Figure 3) through OmpF. Electrophysiology experiments showed that, although avibactam is significantly larger than a simple chloride ion, its permeability through OmpF is comparable. MetaMD simulations provided an explanation for this unexpected result. avibactam showed a number of affinity sites inside the lumen of OmpF, and, correspondingly, FES was characterized by different energy minima with a sharp energy barrier at the CR. Conversely, the FES of Cl^−^ permeation is characterized by a broad energy barrier extending over the entire channel axis (Figure 2). Although energy barrier for avibactam is slightly larger than that found for Cl^−^ (by ca. 2 kcal mol^−^^1^), it is restricted to a specific section of the channel, while Cl^−^ experiences an energy cost all along the translocation process. The detailed analysis of the simulation showed favorable interactions at the POR, as well as deeper in the CR. The minimum energy translocation path of avibactam is characterized by its dipole aligned to the transverse EF of OmpF and, at the same time, direct interactions with both the positive residues of the basic ladder through the sulfate group and with the negatively charged residues of the loop L3 through the amino group. According to our model where the steric barrier to translocation can be compensated by favorable interactions, avibactam showed a remarkably good condition since its relatively small size allows it to fit into the CR with an orientation of its electric dipole well aligned to channel’s EF. In this work, we wanted to extend the investigation to some avibactam derivatives of larger size (Figure 3), in order to assess what key molecular features are important for transport.

While avibactam has an almost spherical shape, all the other selected inhibitors have the amide group functionalized with relatively long substituent. This modification makes one of the three main inertia axes significantly longer than the others, since amide substituent is located opposite to the sulfate group with respect to the diazobicyclooctan. Thus, differently from avibactam, the three selected molecules have an almost cylindrical shape. In addition, the substituent is characterized by one positively charged group, making the molecule zwitterionic with a remarkable dipole moment along the main axis of inertia (Table 2). In Table 2, the inhibitors are listed following the increasing order of molecular volume.

The selected inhibitors are characterized by the average minimal projection area (MPA) and the corresponding average radius ($MPA=\pi R^{2}$) (Table 2) similar to or larger than that of the porins at the CR (Figure 1). Thus, the entropic contribution to the total free energy profile along the translocation path is expected to be remarkable. The steric constraints create a barrier in the CR due to the limitation of the molecular configuration space. Only in selected conformations having a smaller minimal projection area than the average one, the molecule can effectively pass the CR of the porin [55].

All of the investigated avibactam analogues are characterized by a predominant axis of inertia. The electric dipole is mainly due to the presence of the negatively charged sulfate group and a positively charged amino group on the amide substituent. The electric dipole is almost perfectly aligned with the predominant molecular axis of inertia. If the molecule crossed the CR by staying aligned with the channel axis, entropic barrier would be minimum but the electric dipole would be poorly aligned with the channel electric field [48]. On the other hand, if the molecule tried to align the electric dipole to the field lines in the CR, it would have to translocate almost perpendicular to the channel axis and the steric barrier would increase.

**Figure 4:**
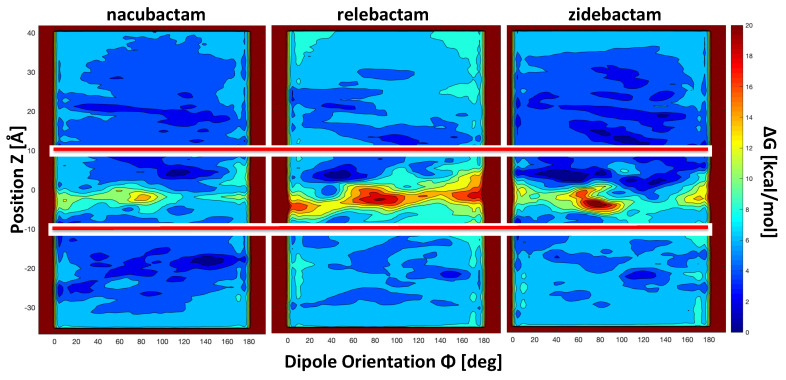
Contour plot of the two-dimensional free-energy surfaces reconstructed from metaMD simulations. Each color corresponds to a free energy difference of 2 kcal/mol. The “position *z*” of the molecule along the channel axis and the “orientation Φ” of the molecule’s electric dipole moment were chosen as collective variables. The two red lines delimit the pre-orientation and constriction regions.

The FES obtained for the permeation of the selected diazobicyclooctans β-lactamase inhibitors through OmpF are shown in Figure 4, as contour plot in the space of the two collective variables. FESs were reconstructed with metaMD by integration and sign inversion of the Gaussian energy-biases added during the simulation.

**Figure 5:**
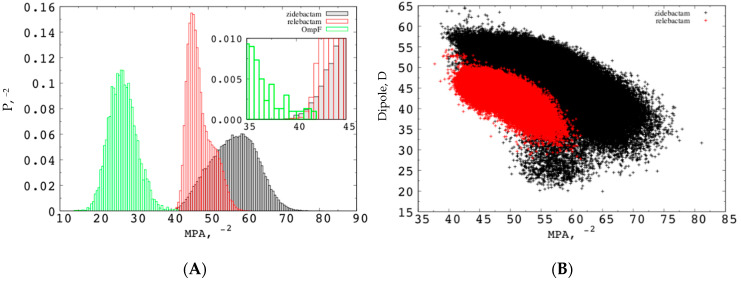
(**A**) Probability distribution of the MPA for zidebactam/relebactam and size of OmpF. (**B**) Scatter plot of dipole moment and minimal projection area for relebactam (red) and zidebactam (black) as sampled during the MD simulations in water.

The “*z*-coordinate” of the center of mass (which corresponds to the position of the substrate along the channel axis with respect to the center of the pore) and the “orientation Φ” of molecule’s dipole moment with respect to the *z*-axis were selected as collective variables. Values of Φ smaller than 90° correspond to the electric dipole pointing upwards (i.e., towards the EV with the positively charged group), values of Φ larger than 90° correspond to the electric dipole pointing downwards (i.e., towards the PV with the positively charged group). The region of *z*-coordinate between 10 Å and −10 Å is the most important one inside the pore as far as the permeation is concerned. This is rich of energy minima, forming the minimum free energy path to cross the central energy barrier at the CR. Not surprisingly, all the inhibitors show the highest free energy values in the most restricted region of the channel, as reported in the literature for a number of antibiotics [42]. The energy barrier extends over the full Φ range for all the investigated inhibitors. In the case of the smallest substrate, nacubactam, the energy barrier appears comparable to the case of avibactam [53], but the effect of increasing size is evident comparing the different height of the energy barrier at the CR (Figure 4). The three FES share one interesting feature of the central energy barrier, which is related to their cylindrical shape: two saddle points with different energy at ca. 40–120°, respectively. It is worth noting that the high Φ saddle point has significantly less energy than the other one in the case of both nacubactam and zidebactam, with a difference of 2 kcal/mol. In the case of relebactam, conversely, it is the low Φ saddle point to be the one with the lowest energy. In addition, it is very interesting to note that, overall, relebactam itself is characterized by the highest energy barrier, instead of the largest inhibitor investigated, zidebactam. These results show that, although molecular weight is a very important parameter in determining the permeation energetics, it is clearly not sufficient, and other physicochemical descriptors are needed [39], such as the MPA. Interestingly, the comparison of the single MPA average value is not conclusive. As shown in Table 2, zidebactam shows a larger MPA average value, as well as larger fluctuations than relebactam, suggesting a different distribution of the MPA. Looking more in detail at the entire distribution of MPA sampled during the MD simulations in water, we see that the apparently larger zidebactam can reach values of the MPA as low as relebactam (Figure 5A), due to the large flexibility of its tail. Interestingly, the smallest region of MPA values in the distribution is also the one that overlap with the largest region of the OmpF pore size, as shown in the inset of Figure 5A.

The other point of attention is the dipole moment. Zidebactam shows an average larger dipole moment that relebactam, suggesting a larger compensation of the steric free energy. The smallest values of the minimal projection area are also characterized by a larger dipole moment of zidebactam with respect to relebactam, as shown by the two-dimension distribution plot (Figure 5B). In fact, the size and the dipole moment are not uncorrelated, as a small minimal projection area (molecules highly extended) corresponds to a large electric dipole moment.

In order to obtain a more detailed description of the interactions between the inhibitors and the protein channel, the conformers were extracted from the energy minima and the saddle point along the minimum energy path on the FES for 10 Å > z > −10 Å. The representative structure for each basin and for the saddle point was obtained through the cluster analysis as the structure of the conformer with the lowest average root mean square deviation (rmsd) from all the other conformers. The analyzed regions of the FES and the representative structures are reported in Supplementary Materials Figure S8, together with the corresponding electric dipole. The smaller size and high flexibility of nacubactam are reflected by the orientation assumed all along the translocation through the CR. Already, from the POR, the molecule tends to align its electric dipole to the channel’s EF lines, and this corresponds to a relatively low energy barrier for permeation. The case of relebactam is particularly unfavorable. The significant larger size, together with the small electric dipole and the reduced plasticity, appear to hinder the molecule from assuming the orientation corresponding to the alignment of the electric dipole with the channel’s EF. The translocation appears to be dominated by the direct electrostatic interaction between the negatively charged sulfate group and the residues comprising the basic ladder, similarly to what was observed in the case of avibactam (whose electric dipole is even smaller due to its small size) [53]. As a consequence, relebactam is forced to translocate with very small Φ angles to minimize the steric hindrance, but it is evident from the large energy of the central barrier that this is not sufficient in the absence of good alignment of the electric dipole with the channel’s EF. Conversely, in the case of zidebactam, in spite of the larger average size, the flexibility of the amide substituent allows zidebactam to adapt better to the pore profile while translocating, and this, together with the presence of a larger electric dipole, clearly drives and enhances the permeation of this inhibitor when compared to relebactam. The long zidebactam needs even to change orientation during the translocation through the CR. Nevertheless, the high free energy barrier expected is clearly compensated by the partial alignment of a larger electric dipole, when compared to that of relebactam, with the channel’s EF.

In summary, in the case of zidebactam, the alignment of the large electric dipole to the channel’s EF represents an effective enthalpic compensation to the entropic barrier, which is allowed by its flexibility. Differently, the smaller electric dipole, together with the considerable rigidity of relebactam, make its FES the one with the largest barrier among the inhibitors investigated in this work.

**Figure 6:**
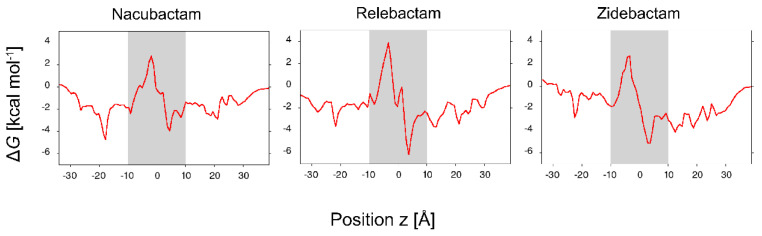
One-dimensional free-energy profiles obtained from the same metaMD simulations of Figure 4, as a function of the “position *z*” of the molecule along the channel axis. The shaded area indicates the pre-orientation and the constriction region.

**Figure 7:**
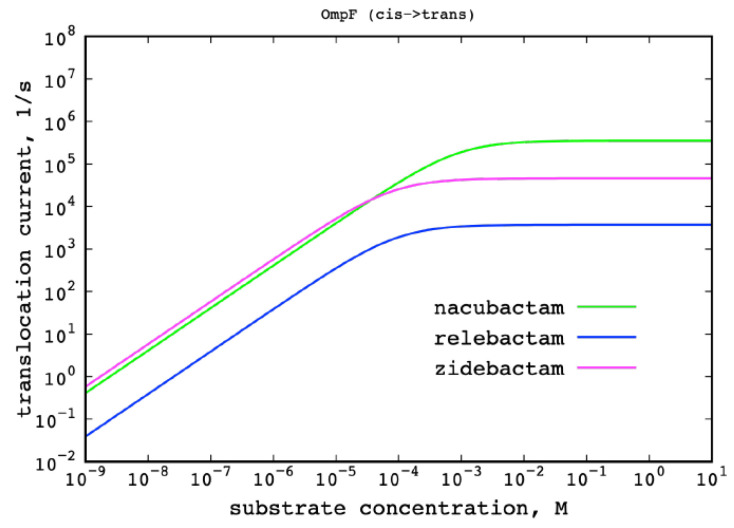
Macroscopic flux of the four investigated inhibitors through the OmpF trimer at different molecules’ gradient concentration.

Additional information can be obtained by comparing the one-dimensional FES shown in Figure 6. These are obtained by weighting the two-dimensional FES of Figure 4 for all orientations and reporting the free energy as function of the substrate *z* position only. While the height of the maximum of the one-dimensional free energies is comparable for the three inhibitors, with the only exception of relebactam, the profile in the shaded region of Figure 6, i.e., the POR and the CR, is rather different. As obtained for avibactam [53], several local energy minima can be observed, whose position and depth depend on the specific inhibitor. A feature common to all the compounds is the presence of remarkable basins around *z* ~5 Å. This corresponds to the POR suggesting that zwitterionic molecules have a greater affinity for the POR, which is the gate to the more restricted CR. Then, among the zwitterionic molecules, the strength of the electric dipole makes the difference in driving the molecule into the favored orientation with respect to the channel’s EF lines, as discussed above [50]. The depth of these minima is not as deep as for substrates permeating specific porins [56]. Instead they have values comparable to the permeation of small substrates through unspecific pores [25]. The free energy, G(x), determines the probability density of a collective variable, x, in the thermodynamic equilibrium, according to the Gibbs distribution, $p\left(x\right)\propto \exp(\;-\frac{G\left(x\right)}{kT})$. Moreover, it also gives the local mean force, $F\left(x\right)=-\frac{\partial G\left(x\right)}{\partial x}$, which, together with the local diffusion coefficient, D(x), determines the facilitated diffusion of the molecule through the pore. At a low substrate concentration, one can neglect the interaction between the substrate molecules. Then, the diffusion is described by the linear 1D Smoluchowski equation, and the diffusion current is proportional to the substrate concentration gradient and is given by a Kramers-type integral formula [53,55]. At a higher substrate concentration, especially in the case of favorable pore–substrate interactions (negative free energy in our normalization), the probability to have two substrate molecules trying to occupy the pore at the same time is not negligible. That leads to the slower growth of the diffusion current vs. the substrate concentration, and finally to the saturation—the independence of the diffusive current on the concentration. To take into account the saturation effect, we have bridged the diffusion-scale model with the two-state Markov model of the particle-pore kinetics [56]. The two-state Markov model assumes that, at the maximum, one particle at a time may occupy the channel. It can describe both the linearity with the concentration and the saturated behavior of the particle translocation regime. We have calculated from the one-dimensional FES (Figure 6) the diffusive current of molecules through a single pore by following References [56], from EV to PV, at different molecule gradient concentrations (Figure 7). At 1 μM gradient concentration, the number of permeating molecules is between 10 (relebactam) and a few hundred (nacubactam and zidebactam). The presence of energy minima for the zwitterionic molecules in the FES gives the current saturation at a concentration above 100 µM, which is quite large with respect to the expected physiological concentrations (<10 µM). It is interesting to note that the saturation occurs at lower concentration for zidebactam than for nacubactam, correlating with a deeper minimum in POR, caused by the larger dipole moment.

The results presented in this work confirm those already reported for avibactam: Ref. [53] The height of the energy barrier is not important per se, but also its width along the channel and the presence of local minima need to be considered. Although the molecular weight is very important for permeation, the distribution of the minimal projection area, which accounts for flexibility, the charge distribution, or the electric dipole, are extremely important parameters that can modulate the permeation of substrates. Moreover, the electric dipole depends on the instantaneous conformation of the molecule, so that also molecular flexibility, together with the distribution of charges, assumes an important role for electrostatic interactions.

## 3. Methods

### 3.1. Molecular Dynamics

X-ray structures of OmpF (PDB ID 2OMF), Omp35 (PDB ID 5O78), OmpE35 (PDB ID 6ENE), OmpK35 (PDB ID 5O77), OmpC (PDB ID 2J1N), Omp36 (PDB ID 5O9C), OmpE36 (PDB ID 5FVN), and OmpK36 (PDB ID 5O79) were used as starting coordinates for molecular dynamics (MD) simulations. All the amino acid residues were simulated in their ionization state at neutral pH, except for E296 (OmpF), D299 (OmpC), E102 (Omp35), D307 (Omp36), D235 (OmpE35), D295 (OmpE36), E102 (OmpK35), and D297 (OmpK36), which were protonated (net charge 0), in all the three monomers for each trimer, as suggested for OmpF by Varma et al [57]. Each trimer was embedded in a pre-equilibrated POPC (1-palmitoyl-2-oleoyl-sn-glycero-3-phosphocholine) bilayer of 259 lipids. The system was oriented in order to center the protein at the origin of the coordinate system and align the channel diffusion axis along the *z*-axis, hence *z* positive values refer to the extracellular vestibule (EV), and *z* negative values refer to the periplasmic vestibule (PV). Using the NAMD software version 2.12, USA [58] the system was equilibrated in the gas-phase, in order to force lipids to adhere the hydrophobic regions of the porins. After 1 ps of energy minimization (conjugate gradients), a slow heating from 10 to 300 K was carried out for 1 ns, with positional restraints on the protein’s alpha carbons along the three dimensions and on the lipids phosphorus atoms along *z* only. The system was solvated with ~17,000 water molecules, and the total number of atoms was ~100 K in a box with size 11 × 11 × 9 nm. A suitable number of potassium and chloride ions were added to reach the desired concentration. An excess of K^+^ ions was required to neutralize the total negative charge of the trimer (−33 e). After releasing the constraints, an equilibration stage of 4 ns in the NPT ensemble at 1.0 bar and 300 K was performed. Finally, 400 ns MD simulations were performed in the NVT ensemble without restraints. Only the last 300 ns were used for structural analysis. Production run in the NVT ensemble was performed by using the ACEMD code [59], running on GPUs, with a time step of 4 fs. This long time step is allowed by the repartitioning of masses between heavy atoms and hydrogens (h − mass = 4 amu) [60]. The Langevin thermostat (300 K) was used with 0.1 ps damping time, and the particle mesh Ewald (PME) method with 9 Å cutoff for electrostatic interactions. The Amber99SB-ILDN force field parameters were used for OmpF, the General Amber Force Field (GAFFlipid) for POPC, and the TIP3P model for waters.

### 3.2. Substrate Parameterization

The Gaff-force field parameters were obtained by following the protocols adopted in http://www.dsf.unica.it/~gmalloci/abdb/ [61]. We used the ChemAxon’s Marvin suite of programs [54] to draw the structure and find the most populated protonation states at physiological pH = 7.4 (Supplementary Materials Figures S8 and S9). The Gaussian09 package [62] was used to perform ground-state geometry optimization of the main tautomer in implicit solvent (Polarizable Continuum Model), by applying the Density Functional Theory (DFT), the hybrid exchange-correlation functional B3LYP, and the 6−31G(d,p) Gaussian basis-set. Atomic partial charges in the optimized conformations were calculated by using Gaussian09 [62] at the HF/6−31G * theory level in vacuum and then generated through the two-step restrained electrostatic potential (RESP) method, as implemented in the Antechamber package. For nacubactam, however, the dominant tautomer at pH = 7.4, suggested by ChemAxon’s Marvin is negatively charged, with a removed proton on the non-terminal nitrogen in the chain, compared with that shown in Figure 3. This latter conformation of the tautomer is not stable (it is not the minimum energy configuration) in the quantum chemical calculations both at the HF/6−31G * and at the B3LYP/6−31G(d,p) theory levels, with and without implicit solvent. Another cheminformatic program, Open Babel v.2.3.2 [63], which also employs an empirical method to assign the main tautomer at a given pH, suggests the zwitterionic form of the nacubactam shown in Figure 3 as the most populated at pH 7.4. This zwitterionic form of nacubactam was verified to be stable in the quantum chemical calculations and was used in the simulations.

Each inhibitor was placed above the first monomer in the EV, about 20 Å away from the constriction region (CR), using the final configuration from the protein simulation.

### 3.3. Enhancing Sampling: Metadynamics

Because the typical time for a substrate to permeate through such a pore is of the order of several hundreds of microseconds, a bias method is necessary to simulate such a process. Substrates permeation was investigated by using well-tempered metadynamics simulations (WT-METAD) [64] with Plumed 2.2 plug-in within the ACEMD software [59]. This method has two features: (i) It works in the reduced space described by a few collective variables, chosen to reproduce the process, and (ii) it modifies the underlying potential by adding repulsive Gaussian terms during the simulations. These bias terms prevent the substrate to visit the same conformations and thus accelerate its evolution in the space of the defined collective variables. In the well-tempered scheme, the Gaussian terms are periodically rescaled, providing a convergence parameter to monitor during the metadynamics simulation [64,65]. When at convergence, the bias potential can be used to reconstruct the underlying free energy surface in the space of defined collective variables.

A first step of normal metadynamics simulation of inhibitor permeation was performed until the first effective translocation through the protein constriction region (CR) was observed. Then, four configurations were selected, two with the inhibitor located in the extracellular vestibule (EV), and two in the periplasmic vestibule (PV). Correspondingly, we performed well-tempered metadynamics with four replicas (also known as multiple-walkers metadynamics) [66], each contributing to reconstruct the same free energy surface (FES).

We defined two collective variables to be able to follow the permeation of substrates through the pore: (i) the substrates “position *z*”, defined as the difference of the *z* coordinate between the center of mass (com) of the substrate and that of the porin first monomer (on the basis of alpha carbons); (ii) the “orientation Φ”, defined as the *z* component of the substrate’s dipole moment. These two geometric variables allow the substrate to sample more easily all positions inside the pore along the axis of diffusion z, by exploring also different orientations.

Each walker was run for at least 450 ns, corresponding to a minimum total simulation time of 1.8 µs. During the metadynamics, the energy biases were added every 5.0 ps to each CV (initial height 1.0 kcal/mol; σ = 5 degree and 0.4 Å for orientation and position, respectively). Well-tempered ∆T was 4800 K. The FES of the translocation process is reconstructed by the integration and sign inversion of the Gaussian bias functions added during a simulation

## 4. Conclusions

We demonstrated with all-atom molecular dynamics simulations that the OmpF/OmpC orthologs from *Enterobacteriaceae* species share common geometrical and electrostatic properties. These properties can modulate the passive permeation of polar molecules, coupling with the size, the flexibility, and the electrostatic properties of the diffusing substrates. In particular, considering a series of avibactam derivatives, all under investigation as potential beta-lactamase inhibitors, we showed that the size of the molecules is better described, in the present context of permeation, by its minimal projection area instead of the molecular mass or volume, taking into consideration its distribution. Furthermore, the exact location of the charged groups in the molecular scaffold, which is described by the strength and direction of the electric dipole moment, is crucial to have an efficient compensation of the free energy barrier due to the decreased size of the pore. Thus, general molecular parameters, such as the minimal projection area and its distribution, the charge, and dipole moment, more than the presence or absence of particular chemical groups, are key to determine the permeation through hourglass-shaped pores such as the main general porins in Gram-negative bacteria. Interestingly, the molecular size and the dipole moment are not independent parameters. This still confirms the possibility to predict the permeability of molecules through porins by using a few molecular parameters, when properly considered.

## Figures and Tables

**Table 1:** Experimental ion selectivity of the OmpF and OmpC orthologs [7].

	OmpF	Omp35	OmpE35	OmpK35	OmpC	Omp36	OmpE36	OmpK36
PK+PCl−	1.3	1.7	1.4	1.4	2.2	2.2	2.1	1.7

**Table 2:** Physicochemical parameters describing the size and electric charge distribution of the β-lactamase inhibitors investigated in this work. The Lewis structures of the compounds are reported in Figure 3. The reported data are average values obtained by sampling the conformations of the molecules in a box of water over Molecular Dynamics with 1 µs simulation time and the corresponding standard deviations. The Van der Waals volumes are calculated by using the ChemAxon’s Marvin suite of programs [54].

	Net Charge at Neutral pH (/e C)	Electric Dipole Moment (D)	Minimal Projection Area (Å2)	Radius of MPA (Å)	Van der Waals Volume (Å3)
avibactam	−1	18 ± 1	43 ± 2	3.7 ± 0.1	199
nacubactam	0 (zwitterion)	46 ± 4	46 ± 3	3.8 ± 0.1	257
relebactam	0 (zwitterion)	44 ± 3	48 ± 3	3.9 ± 0.1	286
zidebactam	0 (zwitterion)	47 ± 6	57 ± 6	4.2 ± 0.2	331

## Supplementary Materials

The following are available online, Figure S1: Simplified phylogenetic tree of enterobacter porins. Figure S2: Beta barrel structure of investigated OmpF ortholog porins. Figure S3: Beta barrel structure of investigated OmpC ortholog porins. Figure S4: Structure alignment of the OmpF orthologs. Figure S5: Structure alignment of the OmpC orthologs. Figure S6: RMSD of proteins along the equilibration trajectory. Figure S7: Charge distribution on porins. Figure S8: Main affinity sites of molecules inside OmpF.
