# Supplementary material for: Permeation of β-Lactamase Inhibitors through the General Porins of Gram-Negative Bacteria
Source: Molecules. 2020 Dec 5;25(23):5747. doi: 10.3390/molecules25235747 (PMC7730927; doi:10.3390/molecules25235747)
Supplement: Supplementary file 1 [file molecules-25-05747-s001.pdf]

Permeation of β-lactamase inhibitors through the general porins of Gram-negative bacteria

Alessandro Pira,[1] Mariano Andrea Scorciapino,[2] Igor V. Bodrenko,[3] Andrea Bosin,[1] Silvia Acosta-Gutierrez,[4] Matteo Ceccarelli,[1,3,]*

[1]Department of Physics and [2]Department of Chemical and Geological Sciences, University of Cagliari, Cittadella Universitaria di Monserrato, S.P.8 km 0,700 – 09042 Monserrato (CA), Italy; [3]CNR/IOM Sezione di Cagliari, Cittadella Universitaria, S.P. 8 km 0,700 – 09042 Monserrato (CA), Italy; [4]Department of Chemistry, University College London - London, United Kingdom.

* To whom correspondence should be addressed

Supplementary Information

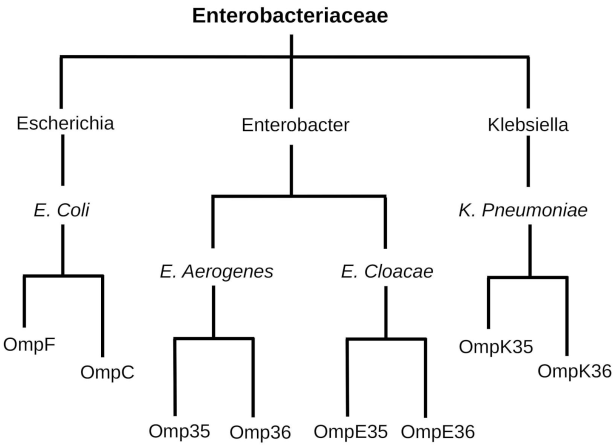

Figure S1. The *Enterobacteriaceae* family if divided into about 30 genera, among which *Escherichia*, *Enterobacter* and *Klebsiella*. In this simplified phylogenetic tree, for each of the four selected species, the two main outer membrane porins are reported. OmpF and OmpC orthologs are on the left- and on the right-hand side, respectively.

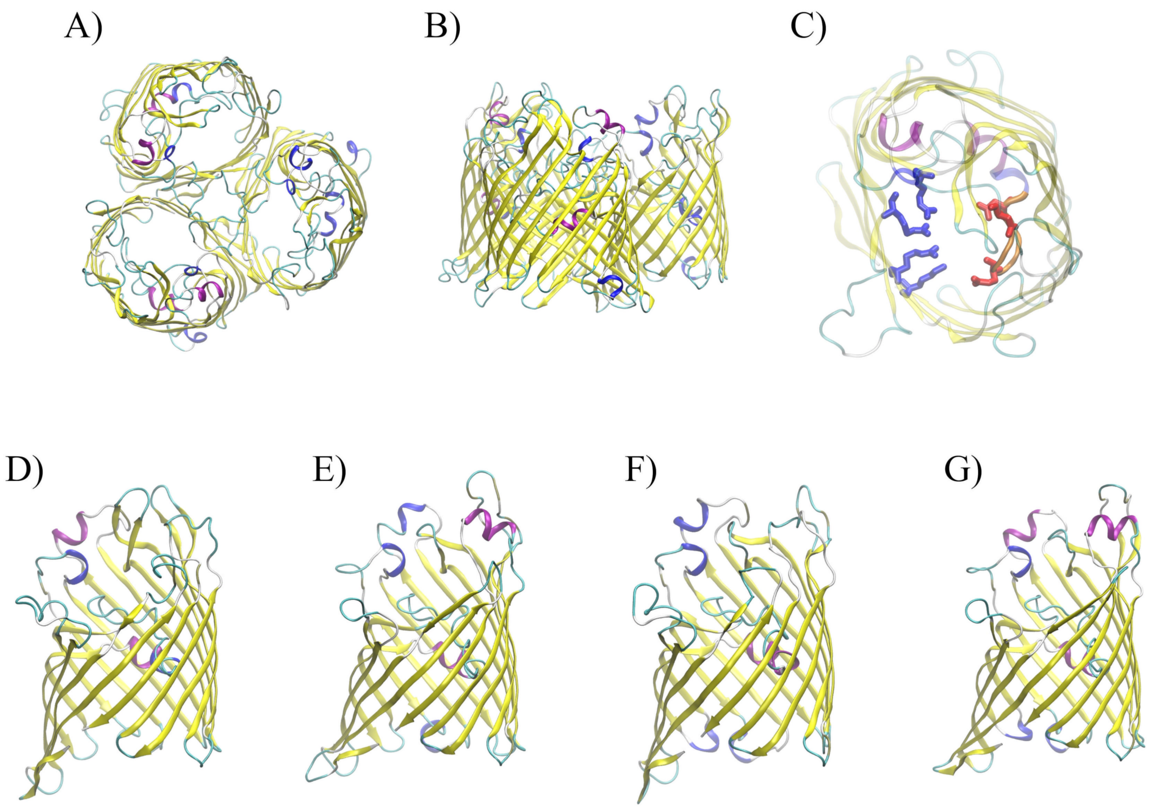

Figure S2. The OmpF trimer is shown, top-view **(A)** and side.view **(B)**. One monomer is shown in **(C)**, top-view, with the loop L3 highlighted, where negatively charged residues are shown in red. On the opposite side of the channel, the positively charged residues comprising the so-called basic ladder are shown in blue. This charge segregation is responsible for the intense electric field at the constriction region (CR). From **(D)** to **(G)**, one monomer (side-view) of OmpF, Omp35, OmpE35 and OmpK35 is shown, respectively.

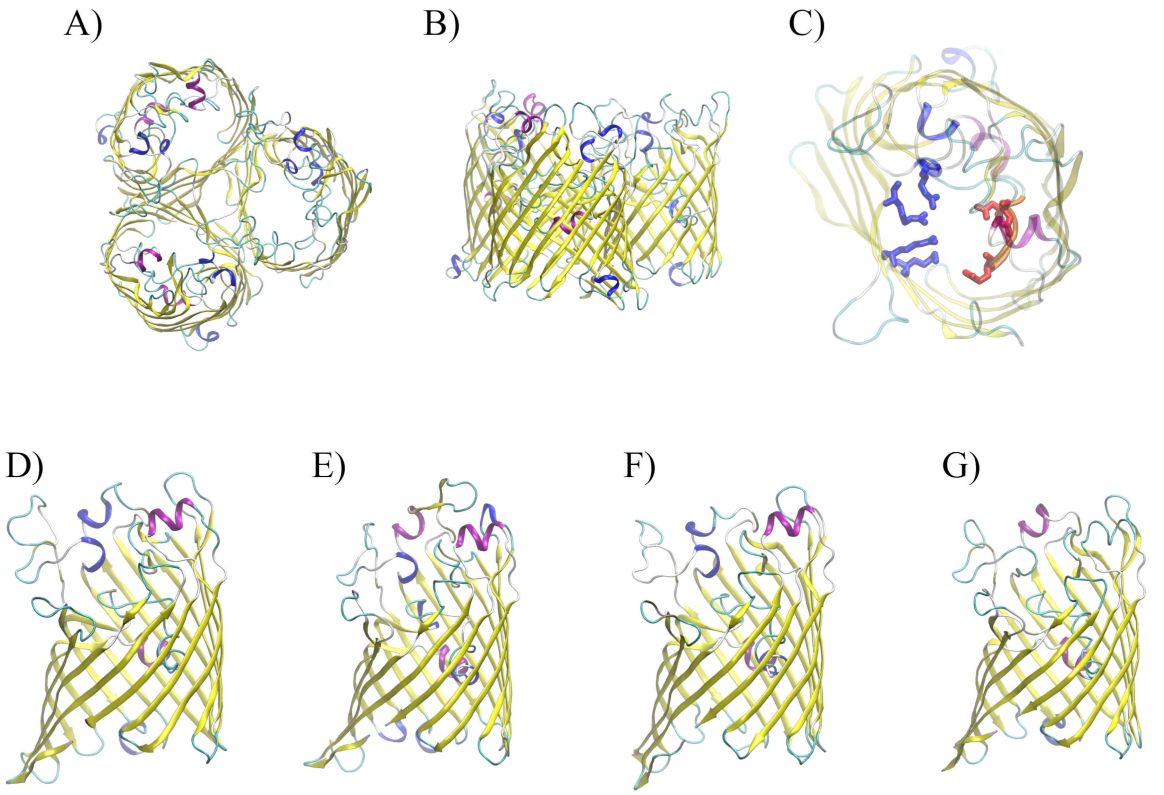

Figure S3. The OmpC trimer is shown, top-view **(A)** and side.view **(B)**. One monomer is shown in **(C)**, top-view, with the loop L3 highlighted, where negatively charged residues are shown in red. On the opposite side of the channel, the positively charged residues comprising the so-called basic ladder are shown in blue. This charge segregation is responsible for the intense electric field at the CR. From **(D)** to **(G)**, one monomer (side-view) of OmpC, Omp36, OmpE36 and OmpK36 is shown, respectively.

```
           β1        β2                 L1          β3           β4
OmpF       AEIYNKDGNK VDLYGKAVGL HYFS-KG--N GENSYGGNGD MTYARLGFKG ETQINSDLTG    57
Omp35      AEIYNKNGNK LDFYGKMVGE HVWTTNGDTS -------SDD TTYARIGLKG ETQINDQLIG    53
OmpE35     AEIYNKDGNK LDLYGKAVGL HYFS-DN--D -G----NDGD KTYARLGFKG ETKINDQLTG    52
OmpK35     AEIYNKNGNK LDFYGKMVGE HVWTTNGDTS -------SDD TTYARIGLKG ETQINDQLIG    53

           β4          L2        β5          β6                L3
OmpF       YGQWEYNFQG NNSEGADAQT GNKTRLAFAG LKYADVGSFD YGRNYGVVYD ALGYTDMLPE   117
Omp35      YGQWEYNMDA SNVEG-SQ-- TTKTRLAFAG LKAGEYGSFD YGRNYGAIYD VESATDMLVE   110
OmpE35     YGQWEYNFQG NNSEGADAQS GNKTRLAFAG LKFGDAGSFD YGRNYGLVYD AIGITDMLPE   112
OmpK35     AEIYNKNGNK LDFYGKMVGE HVWTTNGDTS -------SDD TTYARIGLKG ETQINDQLIG    53

                  L3     β7             β8          L4       β9
OmpF       FGGD-TAYSD DFFVGRVGGV ATYRNSNFFG LVDGLNFAVQ YLGKNERD-T ARRSNGDGVG   175
Omp35      WGGDGWNYTD NFMTGRTNGV ATYRNSDFFG LVDGLSFALQ YQGKNDHDRS IRKQNGDGFS   170
OmpE35     FGGD-TGVSD NFFSGRTGGL ATYRNSGFFG LVDGLNFGVQ YLGKNERT-D ALRSNGDGWA   170
OmpK35     WGGDGWNYTD NYMTGRTNGV ATYRNSDFFG LVDGLSFALQ YQGKNDHDRA IRKQNGDGFS   170

           β9        β10          L5          β11          β12
OmpF       GSISYEY-EG FGIVGAYGAA DRTNLQEAQP LGNGKKAEQW ATGLKYDANN IYLAANYGET   234
Omp35      TAATYAFDNG IALSAGYANS NRSVDQKR-D -GNGDKAEAW ATSAKYDANN IYAAVMYSQT   228
OmpE35     TSLSYDF-DG FGIVGAYGAA DRTNAQQNLQ WGKGDKAEQW ATGLKYDANN IYLAALYGEM   229
OmpK35     WGGDGWNYTD NYMTGRTNGV ATYRNSDFFG LVDGLSFALQ YQGKNDHDRA IRKQNGDGFS   170

                L6          β13           β14             L7     β15
OmpF       RNATPITNKF TNTSGFANKT QDVLLVAQYQ FDFGLRPSIA YTKSKAKDVE G----I--GD   288
Omp35      YNMTPEE--- --DDHFAGKT QNFEAVVQYQ FDFGLRPSLG YVQTKGKNLQ ARGGFGG-GD   282
OmpE35     RNAARLD--- --N-GFANKT QDFSVVAQYQ FDFGLRPSIA YYKSKAKDVE G----I--GD   277
OmpK35     YNMTPEE--- --DNHFAGKT QNFEAVVQYQ FDFGLRPSIG YVQTKGKDLQ SRA-GFSGGD   282

              β15          β16            L8           β1
OmpF       VDLVNYFEVG ATYYFNKNMS TYVDYIINQI DSDNK----L GVGSDDTVAV GIVYQF       340
Omp35      ADLVKYVELG TWYYFNKNMN VYAAYKFNQL DD-NAYTRAA GVATDDQAAV GIVYQF       337
OmpE35     EDYINYIDIG ATYYFNKNMS TYVDYQINQL KDDNK----L GINNDDTVAV GLVYQF       329
OmpK35     ADLVKYIEVG TWYYFNKNMN VYAAYKFNQL DD-NDYTKAA GVATDDQAAV GIVYQF       337
```

Figure S4. Structure alignment of the OmpF orthologs was obtained through the VMD software (https://www.ks.uiuc.edu/Research/vmd/).

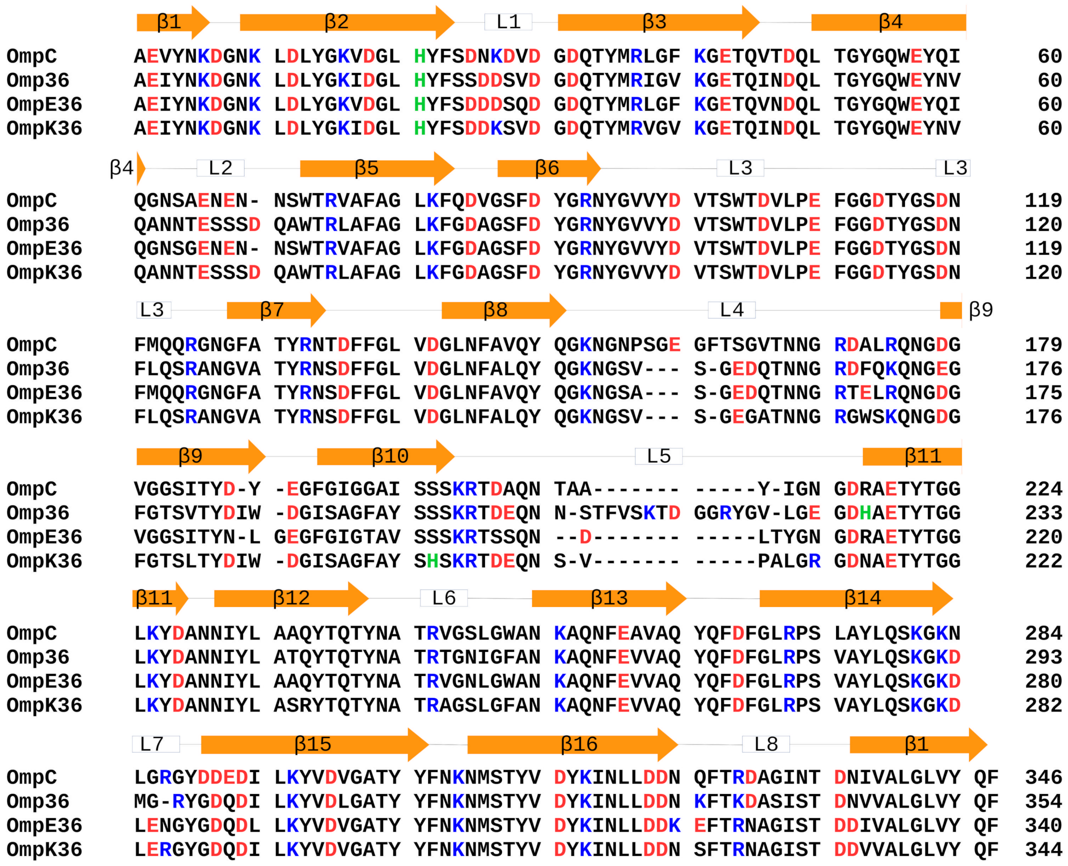

Figure S5. Structure alignment of the OmpC orthologs was obtained through the VMD software (https://www.ks.uiuc.edu/Research/vmd/).

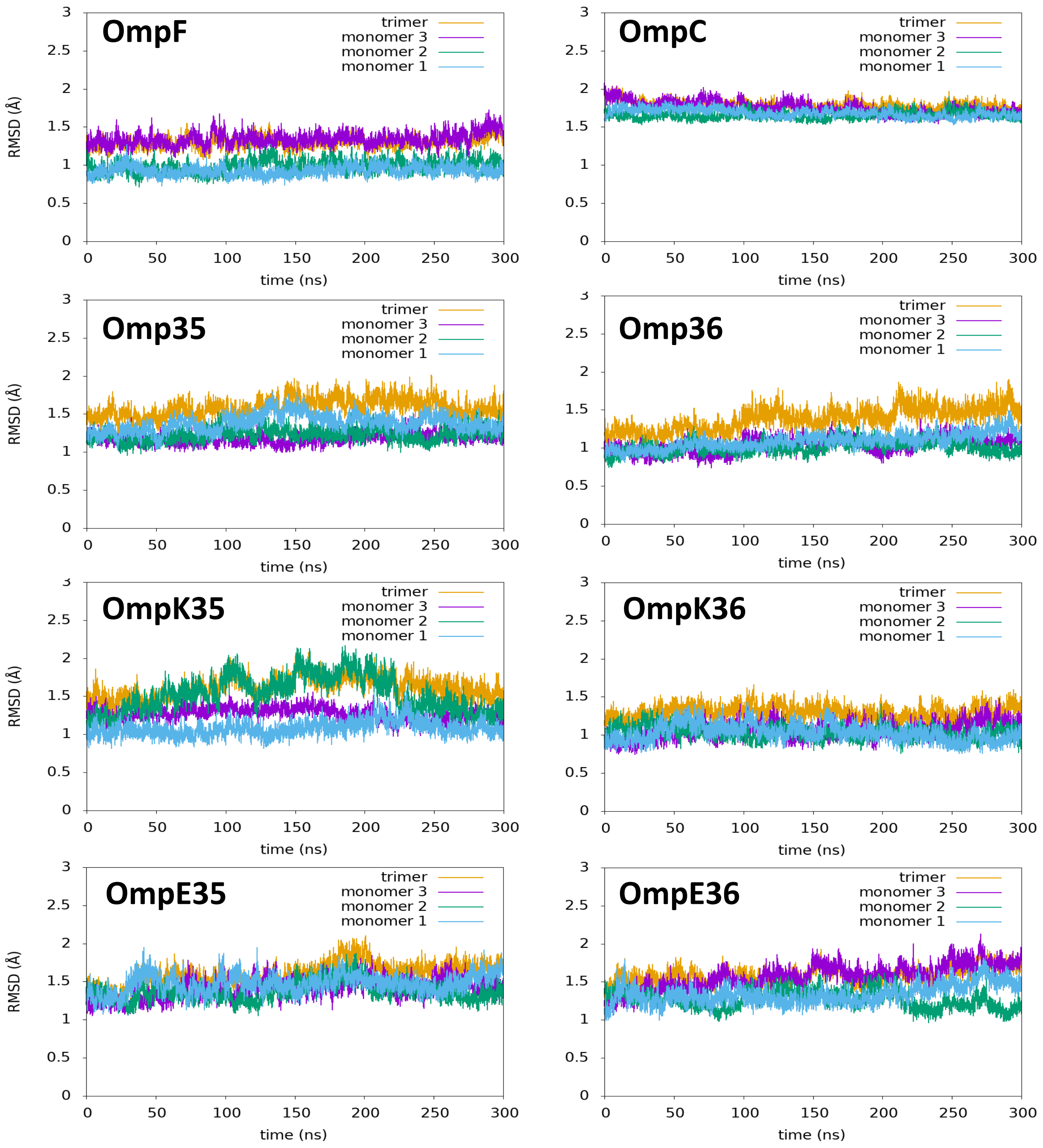

Figure S6 RMSD of the eight porins with respect to the starting X-ray structure, during the last 300 ns of equilibration simulations at 300 K in the NVT ensemble.

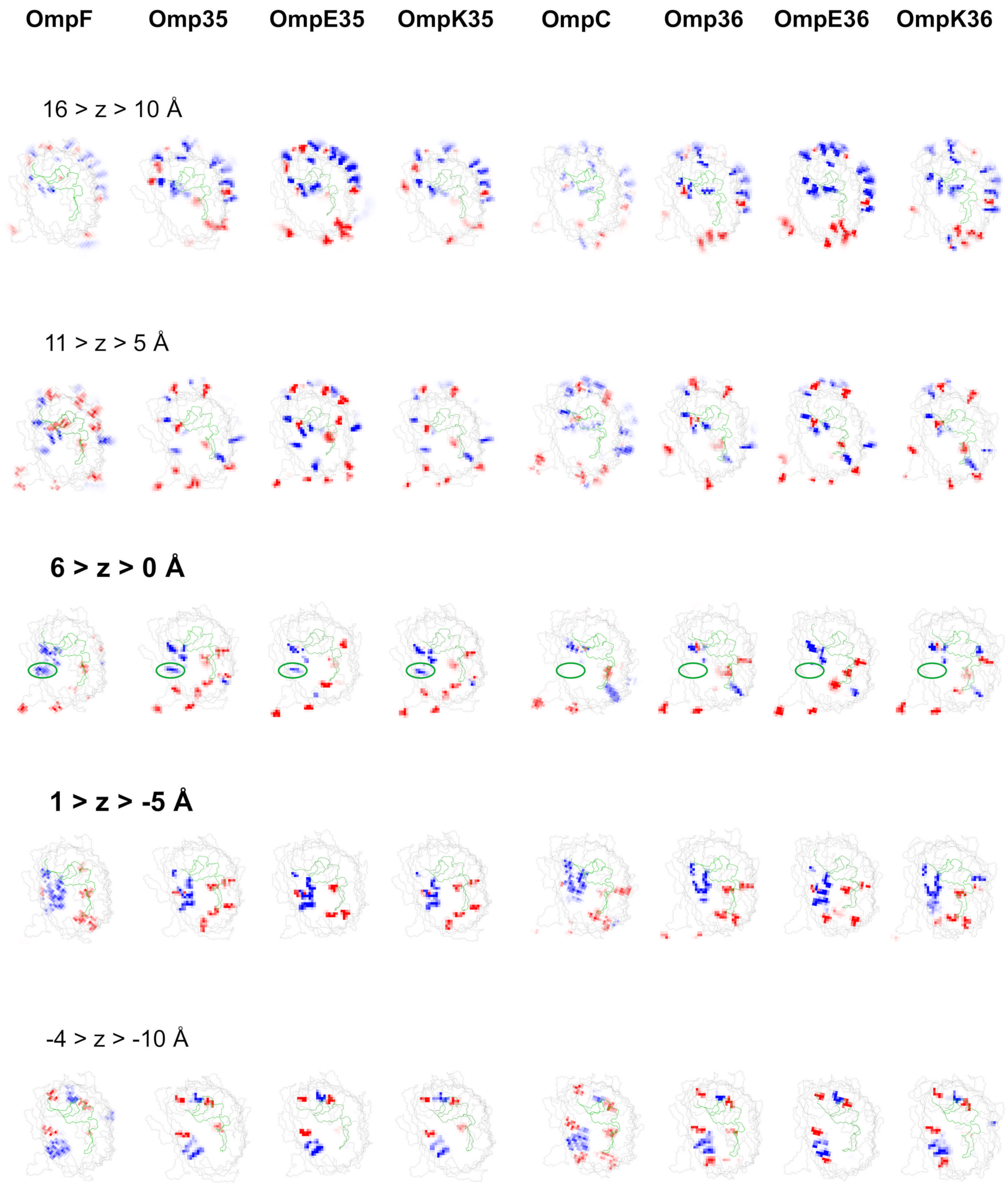

Figure S7. Distribution on the xy-plane of the difference between the density of positively and negatively charged residues, colored in blue and red, respectively. The channel was divided into 5 cross sections along the main axis, namely, +16 Å > z > +10 Å ; +11 Å > z > +5 Å ; +6 Å > z > 0 Å (POR, the pre-orientation region) ; +1 Å > z > -5 Å (CR, the constriction region); -4 Å > z > -10 Å. The circles highlight the major difference found among OmpF orthologs and OmpC orthologs.

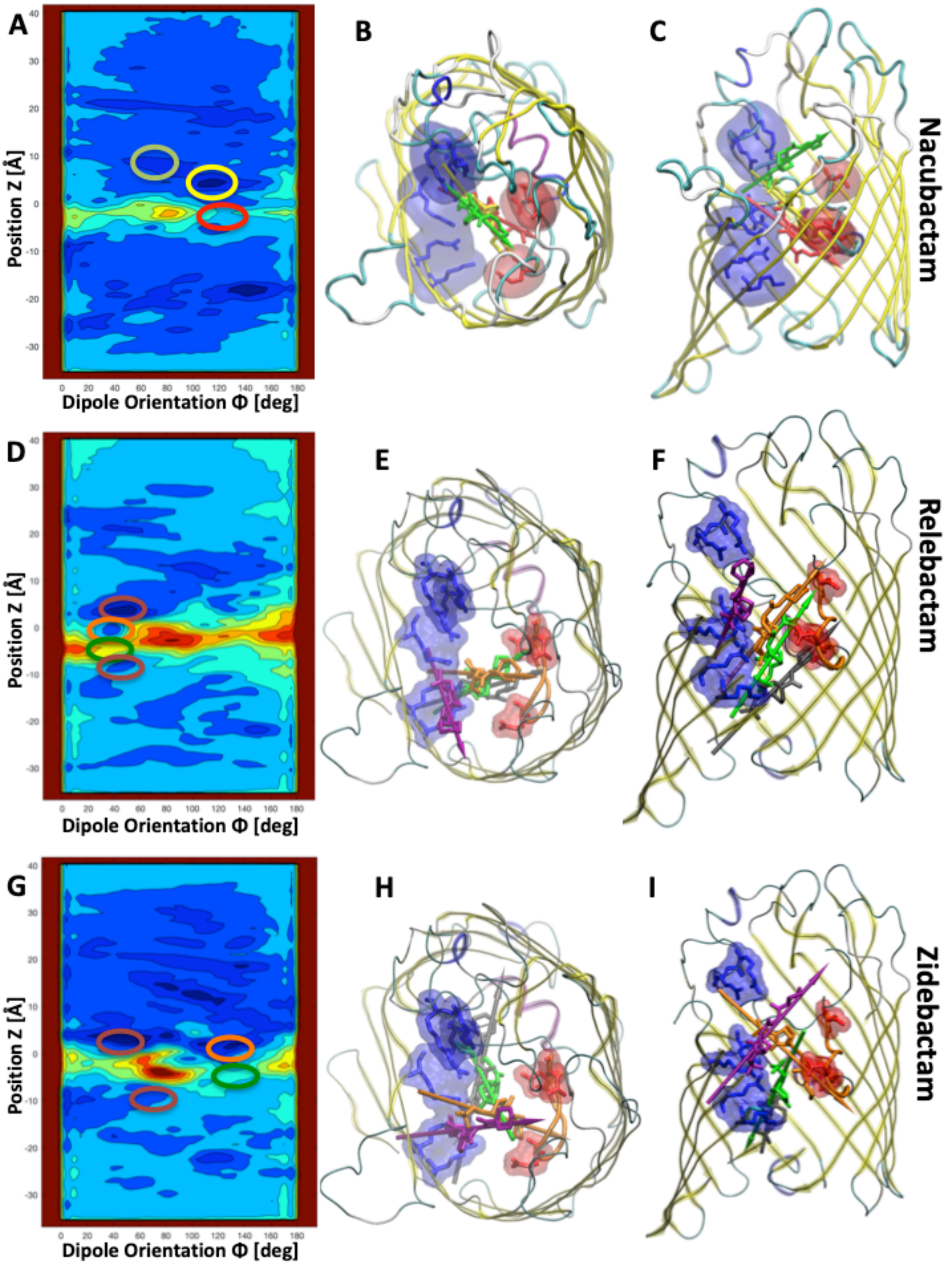

Figure S8. The same FES shown in figure 4 are reported here **(A,D,G)** with differently colored circles to indicate the energy minima and the saddle point analyzed along the minimum energy path in the region 10 Å > z > -10 Å. The same colors are used to show the corresponding representative conformer of the three inhibitors under investigation together with the electric dipole. These structures are shown inside one of the monomers of OmpF from the top view **(B,E,H)** and side view **(C,F,I)**.
